# Rigors

**Published:** 1889-08

**Authors:** W. Gilchrist Burnie

**Affiliations:** Hon. Medical Officer, Bradford Children’s Hospital


### RIGORS: WHAT THEY MAY INDICATE.

By W. Gilchrist Burnie, M. R. C. S.,

Hon. Medical Officer, Bradford Children's Hospital.

In directing attention to the subject entitled as above, and in giving three illustrative cases, I wish to disclaim any intention of attempting (what, indeed, in a paper of this length would be impossible) a thorough inquiry into the important subject of rigors, my sole object being to point out a few of the diseases that rigors may indicate other than those that are now commonly regarded as following them.

The first case is that of a gentleman in comfortable circumstances, to whom I was called at 5 a. m. on Jan. 20th, 1881, and whom I found with right hemiplegia, receiving from his friends the history of an ordinary apopletic attack. The patient was a man aged fifty-six, the son of healthy parents, one of whom attained very old age. He was married, with two grown-up, healthy children. Up to within the past few years he had always enjoyed good health, but had since suffered from stricture of the œsophagus, in consequence of which he had subsisted chiefly on fluid or semi-fluid food. At the time of his attack he was well nourished and in fairly good health. The hemiplegia which was accompanied by paralysis of the bladder pursued an ordinary favorable course, the apparent complete recovery being delayed first by an attack of gout in the toe, and afterwards by a severe attack of cystitis. However, at the end of two months these two complications, together with all paralysis had disappeared, and I felt myself jnstified in giving a favorable prognosis. The patient, however, continued very apathetic, and evinced no desire to get up, although able to do so, and now began to complain of a chilly feeling, and of pain in the right hypochondriac region. This state of affairs lasted until March 30th, when I was hastily summoned one day about noon, to find him in a violent rigor, in which the temperature registered the remarkable height of 107° F. Thereupon I asked my father to see the case with me, which he did, and gave it as his opinion that the patient was suffering from malignant disease of the liver. From this time until the patient's death, which occurred on May 14th, he continued to have one or two severe rigors daily, during which his temperature ranged from 104° to 106°, though in the interim it was normal; and, excepting a very gradual increase in weakness, his symptoms underwent no change. A very marked feature of the case was that, with the exception of the slight constant pain in the region of the liver and the distress during the rigor, the patient made no complaint, and was quite collected up to the last. I should mention that on April 1st Dr. Clifford Allbutt saw the case with me, and that, whilst thinking it to be an obscure one, he did not favor the cancer theory, but gave it as his opinion that the rigors were dependent upon the presence of pus within the abdominal cavity.

On May 16th, two days after the patient's death, necropsy was made. The body was found externally to present no abnormal characteristics. On opening it the cause of death became at once apparent—viz., melanosis. The liver was the organ principally affected, being black throughout, solid, and somewhat enlarged, and on microscopical examination presenting nothing but cancer cells and pigment granules, no liver cells being visible in the specimen observed. The apex of the heart was crowned by a round melanotic mass, the size of a walnut, which was adherent to the pericardium. Spots of melanosis were also found in each kidney, on each of which there was also a cyst, the size of a grape, full of tawny fluid. The spleen was very black and enormously enlarged. No deposits were found in the lungs, although they were distinctly darker than normal. I regret to say that neither the brain nor spinal cord was examined. The œsophageal stricture and pouch, both of which had been diagnosed during life, were discovered. No evidence of disease or cicatrix was met with in the œsophagus, and no pus was found anywhere in the body.

*Remarks.*—I think it will be granted that the trouble it must always be to a general practitioner to perform a post-mortem examination was in the above case amply repaid. Besides the discovery of the cause of death, there were many interesting features about the case: such as the œsophageal stricture and pouch, as well as the perfectly healthy bladder after severe recent purulent catarrh ; and also the fact that the patient, although the victim of such grave and widely-distributed disease, and in spite of his œsophageal stricture, when he died was a well-nourished corpse. I may mention here that during life his heart apparently performed its functions normally, and his urine, on chemical examination, excepting during cystitis, revealed no departure from health. I regret that no microscopic examination of the urine was made, as possibly, had such been done, melanotic granules might have been discovered, and so led to an exact diagnosis. Sir James Paget, I believe, mentions their presence in the urine as often the only sign of the disease. It is also noteworthy, and to my mind instructive, that the disease chiefly affected the excretory organs—the liver and kidneys; and that there was an entire absence of the disease in the skin.

The second case which I desire to call attention to came under my notice when I was a four-year student at St. Bartholomew's Hospital, and made an impression on my mind, not only by its exceptional character, but by the fact that, although from the onset Dr. Andrew, the senior physician to the hospital, had it under his observation together with his then house physician, Dr. West, they were utterly unable, until within a few days of the recovery of the patient from what they regarded as a most grave illness, to venture upon a diagnosis; and I was led to remark to Dr. Andrew that in private practice such a case would be very inconvenient, as the medical man would certainly be expected by the friends of the patients to give a diagnosis. The case was briefly this: A healthy, well nourished young man, after having suffered for a day from general malaise, for which he had given himself a brisk purge, which had acted well, without, however, making him feel any better, went to bed and had a violent rigor, which was followed by very severe pain in the region of the gall-duct. He was now seen by Dr. West, who administered a morphia draught and expressed an opinion that he might be suffering from the passage of a gall-stone. I have not here space to detail this case in full, but it will suffice for my purpose to state that for a fortnight the patient complained of violent frontal headache and pain in the region of the liver, and was subject nightly to elevations of temperature, accompanied by violent rigors, which were followed by most profuse sweats. Constipation was profuse throughout. No purgative was given, it being feared that the case might be one of typhoid fever. Until a few days before the patient's recovery, when Dr. Andrew obtained a history of the syphilis from him, to which disease he attributed the fever, he felt himself unable to do more than say that the man was suffering from a very severe fever, which threatened to prove fatal. After a few days treatment by iodide of potassium the patient made a rapid recovery, and for three or four years enjoyed good health, when syphilitic brain mischief appeared, which caused his death. I should here state that before the syphilitic theory was adopted the formation of an abdominal abscess was regarded as the most probable cause of the rigors.

The third and last case to which I mean to refer was that of a primiparous woman whom I attended in labor last July, and who at the end of a fortnight, when I left home for my annual short holiday, was doing well, but had not yet come downstairs. From this time nothing was heard of the patient till the end of three weeks, when I called on her, and found that after I left she had continued to make good progress until a fortnight previously, when she had a rigor.—After this she had one or two rigors daily, but did not think it necessary to send for me. On examination, I found her pulse to be 120, and her temperature 104°; she also complained of pain in the right iliac region. I at once ordered her upstairs to bed, poultices to be applied, and quinine administered. In a few days, to my surprise she had completely recovered; so I again allowed her to go downstairs and lie upon the sofa. At my next visit another rigor had been experienced, and the fever had returned. Again she was removed upstairs, with the result of the symptoms once more leaving her. It was now that I discovered what was, I believe, the cause of the rigors in this case, viz: a direct communication between the scullery sink (which adjoined the living room) and the sewer. I was led to this discovery by a decided smell of sewer gas in the living-room. The pipe was ordered to be cut off and the patient removed direct from the bed-room into the country, the result being a rapid and complete recovery.

The features in this case to which I would draw attention are: (1) The advent of the rigors and fever immediately on being exposed to sewer gas, and their remarkable departure when removed to an airy upstairs room comparatively free from it. I tremble to think what would have been the result had the labor taken place in the contaminated down stairs room. (2) The fact, that although the rigors continued during a period of three weeks no pus was formed, as was evinced by the complete and rapid recovery of the patient when removed to healthy surroundings.

The chief lesson that the above three cases teach is that we must not look upon the occurrence of repeated rigors in a patient who is not the subject of a malarial fever as of necessity the result of an abscess, or even of a purulent inflammation. A rigor I believe to be simply the danger signal hoisted by nature when a poison enters the blood, or, I ought perhaps rather to say when a poison has not only entered the blood, but has succeeded in so far making good its lodgment as to alter its character. Thus may we regard the initiatory rigor of the specific fevers, where one rigor is generally all that takes place, because the poison only makes one entrance. Thus, in malaria, probably each rigor represents each time that the malarial poison succeeds in modifying the general blood. This takes place, I believe, when rigors occur during the course of a purulent collection upon each entrance of pus into the general circulation, and the same, no doubt, occurred in the three cases narrated. In the first case, probably a rigor occurred at each fresh inoculation from the primary disease in the liver; in the second, at each syphilitic modification taking place in the blood itself, until checked by the iodide of potassium; and in the third, evidently upon each entrance of sewer gas into the already enfeebled puerperal woman. Of course I do not mean that all rigors are due to blood poisoning. Some are of nervous origin, and I well remember a case related by Sir James Paget in a clinical lecture, in which persistent rigors were due to an overlooked urethral stricture. The object of this paper, however, has been to try to prove that one must not too exclusively look upon rigors when they occur apart from the specific fevers and malaria as the indications of pus, but must be on the alert for any blood poison.—*London Lancet.*

